# Overexpression of G0/G1 Switch Gene 2 in Adipose Tissue of Transgenic Quail Inhibits Lipolysis Associated with Egg Laying

**DOI:** 10.3390/ijms17030384

**Published:** 2016-03-15

**Authors:** Paula Renee Chen, Sangsu Shin, Young Min Choi, Elizabeth Kim, Jae Yong Han, Kichoon Lee

**Affiliations:** 1 Department of Animal Sciences, The Ohio State University, Columbus, OH 43210, USA; chen.1930@osu.edu (P.R.C.); kim.4347@osu.edu (E.K.); 2 Department of Animal Biotechnology, Kyungpook National University, Sangju 742-711, Korea; tgsshin@knu.ac.kr; 3 Department of Animal Science and Biotechnology, Kyungpook National University, Sangju 742-711, Korea; ymchoi1@knu.ac.kr; 4 Department of Agricultural Biotechnology, Seoul National University, Seoul 151-921, Korea; jaehan@snu.ac.kr

**Keywords:** G0/G1 switch gene 2, adipose triglyceride lipase, lipolysis, transgenic, Japanese quail

## Abstract

In avians, yolk synthesis is regulated by incorporation of portomicrons from the diet, transport of lipoproteins from the liver, and release of lipids from adipose tissue; however, the extent to which lipolysis in adipose tissue contributes to yolk synthesis and egg production has yet to be elucidated. G0/G1 switch gene 2 (*G0S2*) is known to bind and inhibit adipose triglyceride lipase (ATGL), the rate-limiting enzyme in lipolysis. The objective of this study was to determine whether overexpression of the *G0S2* gene in adipose tissue could successfully inhibit endogenous ATGL activity associated with egg laying. Two independent lines of transgenic quail overexpressing *G0S2* had delayed onset of egg production and reduced number of eggs over a six-week period compared to non-transgenic quail. Although no differences in measured parameters were observed at the pre-laying stage (5 weeks of age), *G0S2* transgenic quail had significantly larger interclavicular fat pad weights and adipocyte sizes and lower NEFA concentrations in the serum at early (1 week after laying first egg) and active laying (5 weeks after laying first egg) stages. Overexpression of *G0S2* inhibited lipolysis during early and active laying, which drastically shifted the balance towards a net accumulation of triacylglycerols and increased adipose tissue mass. Thereby, egg production was negatively affected as less triacylglycerols were catabolized to produce lipids for the yolk.

## 1. Introduction

Adipose tissue is the main storage organ for triacylglycerols (TAG) and can undergo lipolysis to mobilize nonesterified fatty acids (NEFA) in response to stimuli, such as low food intake, heavy exercise, or yolk development in avians [1,2]. Lipolysis is catalyzed by adipose triglyceride lipase (ATGL), which cleaves the fatty acid at the sn-1 position from the glycerol backbone of TAG molecules and is the rate-limiting enzyme in this process [1]. Hormone-sensitive lipase and monoglyceride lipase subsequently hydrolyze the ester bonds of the fatty acids at sn-2 and sn-3, respectively, to result in three NEFAs and glycerol [3]. Recently, G0/G1 switch gene 2 (*G0S2*) was identified as an inhibitor of ATGL in 3T3-L1 adipocytes [4]. *G0S2* is highly expressed in adipose tissue, and the protein contains a conserved hydrophobic domain that binds to the patatin-like domain of ATGL to inhibit its lipolytic activity in adipocytes [5,6,7]. *G0S2* expression has been positively correlated with adipocyte differentiation and accumulation of lipids in several species, including mice, pigs, chickens, and humans [4,5,7,8,9,10].

As follicles mature in the ovary of the hen, lipids originating from the diet as portomicrons, from the liver as lipoproteins, and from adipose tissue as free fatty acids are incorporated into the nutrient-dense yolk [2]. More than half of yolk lipids are in the form of TAG and support about 90% of the energy demands for the developing embryo [11]. A considerable increase in lipid metabolism occurs within the last seven days of incubation, which coincides with the most rapid development of the chicken embryo [12]. The hen, on the other hand, must develop adequate adipose tissue mass to initiate and sustain egg production as demonstrated by markedly increased body weight and fat pad weights of Japanese quail at the onset of laying [2]. During active laying, the quail had a significant decrease in fat pad weights and increased NEFA concentrations in the blood due to high lipolytic activity for mobilizing lipids to the yolk [2].

Although the process of yolk formation has been characterized in poultry, the impact of lipolysis in the adipose tissue for yolk synthesis has yet to be elucidated. Therefore, the current study utilized transgenic quail overexpressing *G0S2* in adipose tissue, which were produced by our lab [13], to determine whether exogenous G0S2 protein could successfully inhibit TAG catabolism associated with egg laying. This may provide insight into an important mechanism of lipolysis regulation for yolk development and egg production in poultry.

## 2. Results

### 2.1. Overexpression of G0S2 Delays Onset of Laying and Reduces Egg Production

*G0S2* transgenic quail from line FG1 began producing eggs about 4.4 days later than non-transgenic FG1 quail (*p* < 0.01), and line FG3 transgenic quail began producing eggs about 5.2 days later than non-transgenic FG3 quail (*p* < 0.01). Over the 6-week period, non-transgenic FG1 quail produced significantly more eggs per week than transgenic FG1 quail (6.29 ± 0.0826 *vs.* 5.9 ± 0.133) (*n* = 18 per group; *p* < 0.05). Likewise, non-transgenic FG3 quail produced significantly more eggs per week than transgenic FG3 quail (6.24 ± 0.0906 *vs.* 5.7 ± 0.107) (*n* = 18 per group; *p* < 0.05). Thus, overexpression of *G0S2* inhibits TAG catabolism in adipose tissue, which is necessary for egg production efficiency.

### 2.2. G0S2 Transgenic Quail Gain Adipose Mass throughout Stages of Laying

**Figure 1:**
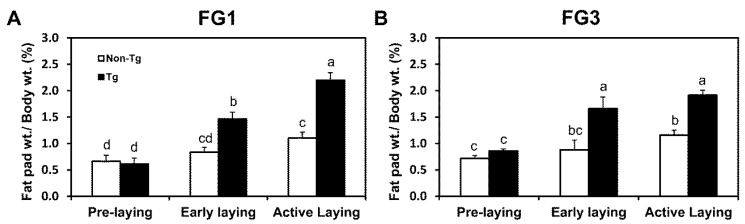
*G0S2* overexpression prevents loss of adipose mass during egg laying. (**A**) Line FG1 and (**B**) line FG3 interclavicular fat pad weights as a percentage of body weights in non-transgenic and transgenic quail at pre-laying, early laying, and active laying (*n* = 5 per group per time point). Bars represent mean ± SEM. Bars that share the same superscript letter are not significantly different from each other (*p* < 0.05).

Interclavicular fat pad weights in non-transgenic and transgenic quail increased after laying; however, fat pad weights drastically increased in transgenic quail when compared to non-transgenic quail as laying progressed (Figure 1A,B). At pre-laying stages, non-transgenic and transgenic quail from both FG1 and FG3 did not have significant differences (*p* > 0.05) in interclavicular fat pad weights, which was expected because of low lipolytic activity. During early laying, FG1 transgenic quail had about 1.8-fold (*p* < 0.05) more subcutaneous fat around the clavicles than FG1 non-transgenic quail, and FG3 transgenic quail had about 1.9-fold (*p* < 0.05) more subcutaneous fat around the clavicles than FG3 non-transgenic quail. Comparison of adipose weights at active laying stages revealed that FG1 and FG3 transgenic quail had about 2.0-fold (*p* < 0.05) and about 1.6-fold (*p* < 0.05) more subcutaneous adipose tissue around the interclavicular region, respectively, than non-transgenic quail. These results demonstrate that *G0S2* transgenic quail continually gain adipose mass as they age, while increases in adipose mass was attenuated by uninhibited lipolysis in non-transgenic quail.

### 2.3. Adipocyte Hypertrophy Caused by G0S2 Overexpression after Onset of Laying

**Figure 2:**
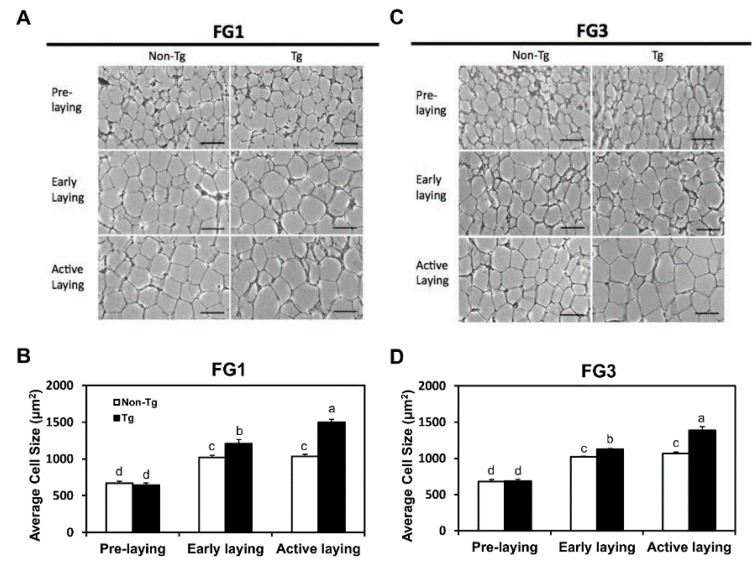
Adipocyte size continually increases *G0S2* transgenic quail, but stays constant in non-transgenic quail during laying. Histological images of adipocytes at different stages of egg laying for (**A**) line FG1 and (**C**) line FG3. Scale bar = 50 μm. Average cell size was calculated for (**B**) line FG1 and (**D**) line FG3 (*n* = 5 per group per time point). Bars represent mean ± SEM. Bars that share the same superscript letter are not significantly different from each other (*p* < 0.05).

Adipose tissues were sectioned, and cell size was measured to determine whether differences in adipose mass between non-transgenic and transgenic was due to adipocyte hypertrophy. No difference in fat cell size between non-transgenic and transgenic quail was observed at pre-laying as expected from the similar interclavicular fat pad weights (Figure 2A,B). At early and active laying stages, FG1 and FG3 transgenic quail had larger adipocytes than the non-transgenic quail. In FG1, transgenic quail had 1.2-fold (*p* < 0.05) larger adipocyte sizes at early laying and 1.5-fold (*p* < 0.05) larger adipocyte sizes at active laying than non-transgenic quail (Figure 2C). In FG3, transgenic quail had 1.1-fold (*p* < 0.05) larger adipocyte sizes at early laying and 1.3-fold (*p* < 0.05) larger adipocyte sizes at active laying than non-transgenic quail (Figure 2D). Adipocytes sizes in non-transgenic and transgenic quail increased after laying initiated, but adipocyte sizes from transgenic quail continued to increase as laying progressed. Increased fat pad weights observed in FG1 and FG3 transgenic quail at early and active laying stages was attributed to adipocyte hypertrophy through *G0S2* overexpression in adipose tissue.

### 2.4. Lipolysis Initiated by Egg Laying Is Inhibited by G0S2

**Figure 3:**
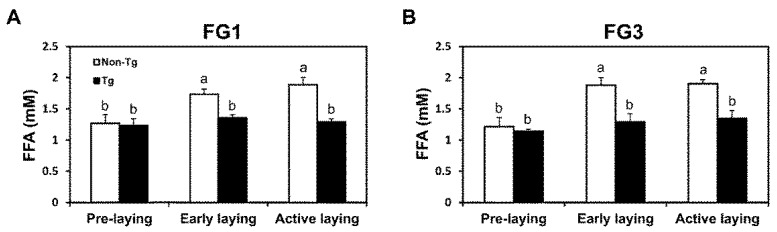
Lipolytic activity due to egg laying is lowered by *G0S2* overexpression. Blood NEFA levels of (**A**) line FG1 and (**B**) line FG3 non-transgenic and transgenic quail at different stages of laying (*n* = 5 per group per time point). Bars represent mean ± SEM. Bars that share the same superscript letter are not significantly different from each other (*p* < 0.05).

No differences in NEFA concentrations in the blood serum (*p* > 0.05) were evident between non-transgenic and transgenic quail from FG1 and FG3 at pre-laying. Transgenic quail from FG1 had 22% (*p* < 0.05) lower NEFA concentrations at early laying and 32% (*p* < 0.05) lower NEFA concentrations in the blood at active laying (Figure 3A). Transgenic quail from FG3 had 31% (*p* < 0.05) lower NEFA concentrations at early laying and 29% (*p* < 0.01) lower NEFA concentrations at active laying (Figure 3B). In non-transgenic quail, NEFA concentrations increased after laying, while transgenic quail did not demonstrate this elevation from pre-laying to early or active laying due to overexpression of *G0S2* blocking lipid mobilization.

**Figure 4:**
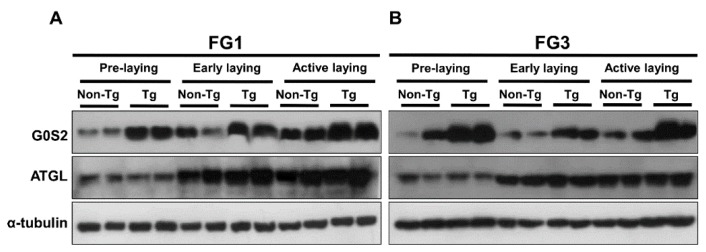
Protein expression of G0S2 and ATGL is altered in transgenic quail. Expression of G0S2 and ATGL in adipose tissue of (**A**) line FG1 and (**B**) line FG3 with α-tubulin as a reference. Two non-transgenic and two transgenic quail from each stage of laying were randomly selected to demonstrate the different expression patterns of G0S2 and ATGL between the groups and across laying stages.

Western blot analysis of G0S2 and ATGL expression revealed that FG1 and FG3 transgenic quail at all laying stages had higher expression of G0S2 compared to non-transgenic quail, whereas ATGL expression in non-transgenic and transgenic quail was higher at early and active laying stages than pre-laying (Figure 4A,B). Thus, lipolysis is normally up-regulated after the onset of laying to mobilize lipids towards the yolk, but overexpression of *G0S2* in adipose tissue of transgenic quail blocks TAG catabolism as evidenced by lower NEFA concentrations in the serum.

## 3. Discussion

Adipose tissue undergoes lipolysis, which is catalyzed by ATGL, to mobilize fatty acids from stored TAGs for yolk synthesis [2]. G0S2 directly binds and inhibits ATGL [4,7]; however, the impact of G0S2 expression on the physiological state of the hen and her egg production has not been extensively studied, as lipolysis is critical for yolk formation. In the current study, we utilized two lines of transgenic Japanese quail overexpressing *G0S2* in the adipose tissue that were previously produced in our lab [13] to investigate the consequences of blocking TAG catabolism at different stages of laying. Our current results support our previous study with *G0S2* transgenic quail, which determined that exogenous G0S2 blocked lipolysis during feed restriction but had no effect on fat accretion during ad libitum feeding [13]. Obvious changes in the hen’s physiological state occur as she transitions into the onset of laying, which is the period of follicular development and yolk deposition before the first egg is laid. As shown by our previous study [2], adipose weights of quail more than double from pre-laying to onset of laying, demonstrating that the hen is readily accumulating TAGs to sustain continuous lipid transfer to the yolk. ATGL expression was low at the onset of laying, which allows for rapid TAG synthesis; however, expression rises during active laying and is concurrent with a reduction in fat cell size and rise in NEFA concentrations in the blood. In the current study, overexpression of *G0S2* resulted in delayed onset of laying and reduced egg production, which is most likely because TAG catabolism was inhibited in adipose tissue that is essential for mobilizing lipids to the yolk. Therefore, onset of laying is delayed and yolk development is slower, which reduces the number of eggs produced by the transgenic hen. Non-transgenic quail from FG1 and FG3 already had significant lipid transfer from the adipose tissue at 1 week after laying the first egg as indicated by lower adipose weights around the clavicles and increased NEFA concentrations. Thus, regulation of ATGL is crucial for production efficiency of the laying hen and controls the timing of yolk development.

Both external and internal factors affect egg production. External factors are environmental and include housing, ambient temperature, season, and feeding conditions, while internal factors are related to the genotype of the hen [14]. Increased energy intake was positively correlated with increased egg production and hen weight [15,16], and feed restriction resulted in decreased egg production [17]. The hens utilize some of the excess energy to build adipose reserves [16], which continually supports egg production through sustained lipid mobilization. Transgenic *G0S2* quail in this study have an altered genotype that inhibits the mobilization of lipids from the adipose tissue into the yolk. Therefore, these quail must rely more substantially upon the diet and liver to contribute lipids for yolk development.

Obesity in humans is directly associated with higher estrogen concentrations, which results in earlier menarche [18,19]. Moreover, these high estrogen concentrations are the product of up-regulated aromatase, converting testosterone to estrogen, in adipose tissue of overweight or obese individuals [20]. Regarding avians, higher estrogen concentrations trigger a metabolic shift that initiates the production of yolk-targeted VLDL from the liver [21,22]. Additionally, the capability to mobilize lipids from the adipose tissue is another main factor that supports the onset of egg production as well as sustained production [2,11]. During yolk formation, lipids are readily incorporated from the adipose tissue, liver, and diet [2], whereas, mammals continuously provide nutrients to the fetus through the placenta [23], which does not warrant the need for large mobilizations of lipid stores. Therefore, sufficient lipid stores within adipose tissue of avians are necessary for initiating and sustaining egg laying.

In summary, overexpression of *G0S2* in the adipose tissue of female Japanese quail was able to inhibit TAG catabolism associated with egg laying. Delayed onset of laying and reduced egg production noted in the transgenic quail was the result of G0S2 blocking TAG catabolism for lipid mobilization to the yolk, and these hens had larger adipose weights and lower NEFA concentrations in the blood. Overall, regulation of ATGL and G0S2 expression in the adipose tissue of the hen is critical for mobilizing lipids to support egg production, implying the regulation of lipid metabolism in adipose tissue is linked to yolk development and consequent reproduction in avians.

## 4. Experimental Section

### 4.1. Animal Use and Ethics Statement

All experimental procedures were conducted in agreement with a protocol approved by the Institutional Animal Care and Use Committee (IACUC) of The Ohio State University (Protocol 2010AG0005) and the Prevention of Cruelty to Animals Act (1986). Two lines of *G0S2* transgenic Japanese quail (Coturnix coturnix japonica), FG1 and FG3, were previously produced in our lab and maintained at the poultry center at The Ohio State University. Fatty acid binding protein 4 (FABP4) promoter was used to direct adipose-specific expression of the *G0S2* transgene. At least two independent transgenic lines were required for this study to ensure the phenotypes were due to the transgene expression and not the integration of the transgene into the genome. Thus, these two lines were selected based on their high expression of *G0S2* in the adipose tissue [13]. Only female *G0S2* transgenic and non-transgenic quail from FG1 and FG3 were used for the current study, and they were fed a standard hen’s diet *ad libitum*.

### 4.2. Detection of G0S2 Transgene by PCR

Genomic DNA from feather pulp and blood from progeny of FG1 and FG3 was extracted at 2 weeks post-hatch and subjected to genotyping PCR. Pulp from one feather was incubated in 300 μL of cell lysis buffer (200 mM NaCl, 50 mM Tris-Cl, 10 mM EDTA, 1% SDS, pH 8.0) containing Proteinase K (0.1 mg/mL, Invitrogen, Carlsbad, CA, USA) at 55 °C for at least 2 h. Blood was extracted from the wing vein with 5 μL of 50 mM EDTA to prevent clotting, and 4 μL was incubated in 300 μL of cell lysis buffer at room temperature for at least 3 h. To remove protein, 300 μL of phenol-chloroform-isoamyl alcohol (PCI) was added, vortexed, and centrifuged at 15,000× *g* for 2 min. The supernatant was transferred to a new tube, and DNA was precipitated by adding 300 μL of isopropanol, inverting, and centrifuging at 15,000× *g* for 5 min. The pellet was washed with 70% ethanol, dried, and dissolved in TE buffer containing RNase A (10 μg/mL, Qiagen, Valencia, CA, USA). The forward primer, LTG0S2-F: 5′-GAGAAGAGACCGAGCCCATC-3′, and reverse primer, WPRE-R: 5′-AAGGGAGATCCGACTCGTCT-3′, were used for genotyping PCR for feather pulp and blood genomic DNA to determine if progeny were transgenic with the positive 798 bp fragment or non-transgenic with no amplification. Positive offspring were reconfirmed with the primer set, LTG0S2F and LT-R: 5′-CGGGCCACAACTCCTCATAA-3′ for the reverse primer, which amplified a 412 bp fragment if transgenic.

### 4.3. Egg Production and Tissue Collection

Onset of laying and number of eggs produced over a 6-week period were recorded for 18 transgenic or non-transgenic quail from FG1 and FG3. To investigate the function of *G0S2* during different laying stages, five female quail from the same parents of either FG1 or FG3 were used for the transgenic or non-transgenic groups based on genotyping. Non-transgenic and transgenic quail adipose and blood was collected at 5 weeks of age (pre-laying), 1 week after laying the first egg (early laying), and 5 weeks after laying the first egg (active laying). Body weight was measured before sacrifice by CO2 inhalation. After sacrifice, subcutaneous adipose tissue from the interclavicular region was excised and weighed. A portion was fixed in 10% neutral buffered formalin (Sigma-Aldrich, St. Louis, MO, USA) for 48 h and replaced with 70% ethanol for histological analysis. The remaining adipose tissue was immediately stored at −80 °C for further analyses. Blood was collected by cardiac puncture and centrifuged at 2000× *g* for 10 min at 4 °C. Serum was transferred to a new tube and stored at −80 °C for non-esterified fatty acids (NEFA) assay.

### 4.4. Histological Processing for Determining Cell Size

All procedures, including dehydration, embedding, sectioning, and staining with hematoxylin and eosin, for the fixed adipose samples from non-transgenic and transgenic FG1 and FG3 quail were conducted in accordance with our previous report [24]. Images were captured with an AXIO-Vert.A1 optical microscope (Carl Zeiss Microscopy, Thornwood, NY, USA) furnished with an AxioCam MRc5 camera (Carl Zeiss Microscopy). All images were analyzed with ImageJ software (NIH ImageJ 1.47, http://imagej.nih.gov/ij) to determine average cell size. Average adipocyte size was calculated by taking the area of a large portion of cells and dividing this by the total number of cells found within the area. At least 1000 cells were analyzed per animal.

### 4.5. NEFA Assay

Serum NEFA concentrations were determined by using the Non-Esterified Fatty Acids Detection 500 Point Kit (Zen-Bio, Inc., Research Triangle Park, NC, USA). All procedures were conducted according to the manufacturer’s protocol. After adding standards, 5 μL of sera, and dilution buffer up to 50 μL into a 96-well plate, 100 μL of FFA Reagent A was added to each well, mixed, and placed in a 37 °C incubator for 10 min. Then, 50 μL of FFA Reagent B was added to each well, mixed, and placed in a 37 °C incubator for 10 min. The plate was equilibrated to room temperature, and optical density was measured at 540 nm. A linear standard curve was generated using Microsoft Excel (Microsoft, Redmond, WA, USA), and each NEFA sample concentration was calculated based on the curve. The 10× dilution of the sera was taken into account for all samples.

### 4.6. Western Blotting

Procedures for Western blot were performed as described in our previous report [25]. Briefly, proteins were isolated from adipose tissues and separated on SDS-PAGE with gel percentage depending on target protein size. Proteins were electrophoretically transferred onto polyvinylidene difluoride (PVDF) membranes (Amersham Biosciences Hybond-P; Amersham Biosciences, Piscataway, NJ, USA) and blocked in 4% nonfat dry milk in 1× Tris-buffered saline containing 0.05% Tween-20 (TBST) for 40 min. G0S2 protein was probed with a rabbit anti-chicken *G0S2* antibody (1:3000 dilution) that was developed in our laboratory [13]. ATGL protein was probed with a rabbit antibody against human ATGL (1:1000 dilution; Cell Signaling Technology, Danvers, MA, USA). After incubating in the respective primary antibody at 4 °C overnight, membranes were washed with 1× TBST and incubated in anti-rabbit IgG antibody conjugated with horseradish peroxidase (HRP; Jackson ImmunoResearch, West Grove, PA, USA) for 1 h at room temperature. Alpha-tubulin protein as a reference was determined with an alpha-tubulin antibody (12G10; Developmental Studies Hybridoma Bank, Iowa City, IA, USA) and an anti-mouse IgG antibody conjugated with HRP (Cell Signaling Technology). Following the next wash with 1× TBST, Amersham ECL plus Western Blotting Detection Reagents (GE Healthcare Biosciences, Pittsburgh, PA, USA) were applied to the membrane and developed on Hyperfilm (GE Healthcare Biosciences).

### 4.7. Statistical Analysis

Differences in means for average eggs produced per week and age of first egg were analyzed by Student’s t-test with *p* < 0.05 declared significant. Descriptive statistics for interclavicular fat pad percentage of body weight, adipocyte sizes, and NEFA concentrations were calculated using the MEANS procedure of SAS (SAS Institute, Cary, NC, USA). Differences between means for non-transgenic and transgenic birds and among laying stages was assessed using a general linear model. Significant differences were detected at a level of 5% by probability difference (PDIFF). Results are presented as least square means with standard errors of the means.

## Abbreviations

The following abbreviations are used in this manuscript:

TAG: triacylglycerol
NEFA: nonesterified fatty acid
ATGL: adipose triglyceride lipase
*G0S2*: G0/G1 switch gene 2
FABP4: fatty acid binding protein 4
